# How and when to use common biomarkers in community-acquired pneumonia

**DOI:** 10.1186/s41479-016-0017-7

**Published:** 2016-10-28

**Authors:** Erica J. Shaddock

**Affiliations:** 1 Division of Pulmonology and Critical Care, Department of Internal Medicine, Area 552 Charlotte Maxeke Johannesburg Academic Hospital, Jubilee Road, Parktown, Johannesburg, 2193 South Africa; 2 Faculty of Health Sciences, University of the Witwatersrand, Johannesburg, South Africa

**Keywords:** Biomarkers, Pneumonia, Procalcitonin, C-reactive protein

## Abstract

Community-acquired pneumonia (CAP) is a leading cause of death in both the developed and developing world. The very young and elderly are especially vulnerable. Even with appropriate early antibiotics we still have not improved the outcomes in these patients since the 1950s, with 30-day case fatality rates of between 10–12%. Interventions to improve outcomes include immunomodulatory agents such as macrolides and corticosteroids. Treating doctors identify CAP patients who are likely to have poor outcomes by using severity scores such as the pneumonia severity index and CURB-65, which allows these patients to be placed in ICU settings from the start of the admission. Another novel way to identify these patients is with the use of biomarkers. This review illustrates how various biomarkers have been shown to predict mortality, complications and response to treatment in CAP patients. The evidence using either procalcitonin or C-reactive protein to demonstrate response to treatment and hence that the antibiotics chosen are appropriate can play an important role in antibiotic stewardship.

## Background

The first description of pneumonia has been credited to Hippocrates, and in the 2500 years following his account we have accumulated vast knowledge and understanding of Osler’s “Captain of the Men of Death”. Community-acquired pneumonia (CAP) is, however, still a leading cause of death in the developed and developing world. Whilst treatment options and diagnostic techniques have improved, overall 30-day mortality for CAP is still 10–12.1% [1, 2], and researchers continue to try to find methods to improve outcomes. One of these approaches is the use of biomarkers and there is a growing body of evidence to suggest that biomarkers could help with this challenge. There are many excellent biomarker review articles available; this review hopes to add to the literature with some recent practical data [3–6].

Biological markers, more commonly called biomarkers, were (as recently as 1998) defined by the National Institutes of Health Biomarkers Definitions Working Group, as “a characteristic that is objectively measured and evaluated as an indicator of normal biological processes, pathogenic processes, or pharmacologic responses to a therapeutic intervention” [7]. Numerous biomarkers have been tested and validated for use in CAP. This review will be examining recent evidence and how biomarkers may be of use in daily practice.

Traditional biomarkers such as white cell count (WCC) and erythrocyte sedimentary rate (ESR) have become less relied upon due to their lower sensitivity and specificity compared to the more promising C-reactive protein (CRP) and procalcitonin (PCT), which are currently in widespread use. Other inflammatory mediators such as interleukin (IL)-1β, IL-6, tumor necrosis factor (TNF)-α, and IL-8, have also been found to be elevated in response to the infection. Unfortunately these pro-inflammatory cytokines have very short half-lives and lack specificity; therefore, they are not currently viewed as good prospective biomarkers [3].

## Basic science

The biomarkers that have been the most thoroughly validated in CAP and that are accessible to most medical practitioners are CRP and PCT.

CRP is secreted from hepatic cells in response to elevated IL-6, IL-1β, and TNF-α. These cytokines are potent pro-inflammatory agents that are released from various innate inflammatory cells when they encounter pathogen-associated molecular patterns (PAMPS) from invading organisms. Additional sources of CRP synthesis have been recently identified and include lymphocytes, monocytes, neurons, and atherosclerotic plaques [8]. CRP’s name originates from its ability to precipitate C-polysaccharide of *Streptococcus pneumonia*, and it was one of the first acute phase proteins to be described. Due to the main production of CRP occurring in the liver, it is important to remember it is not a reliable marker for sepsis in patients with liver failure. CRP synthesis starts very rapidly after a single stimulus: serum concentrations rise within about 6 h and peak around 48 h. The plasma half-life of CRP is about 19 h, which is constant in health and disease. Therefore, the main determinant of plasma CRP concentration is the synthesis rate, which directly reflects the degree of the pathological process stimulating CRP production [8].

PCT is a 116 amino acid peptide, with a molecular weight of 14.5 kDa. It belongs to the calcitonin superfamily of peptides, and is a precursor to calcitonin production usually taking place in the C cells of the thyroid gland [9]. PCT synthesis is very interesting and occurs in a tissue-specific manner. If there is no infection, transcription of the *CALC-1* gene for PCT in the non-neuroendocrine tissue is suppressed, except in the C cells of the thyroid gland. A microbial infection induces a substantial increase of *CALC-1* gene expression in all parenchymal tissue and differentiated cell types in the body that produce PCT. We are currently uncertain of the exact function of PCT synthesised in the non-neuroendocrine tissues under microbial infection [9]. PCT is detectable within 2–4 h of infection, peaks within 6–24 h, and can be present for up to 7 days. The half-life is 22–26 h in plasma and it is cleared mainly through proteolysis with minimal renal excretion [10]. The response to antibiotics can also be monitored well with PCT; as a “rule of thumb”, a decline of > 30% per day indicates improvement of systemic inflammation. This decrease is due to the natural plasma disappearance rate of PCT if no further inflammatory activation is occurring [11]. Interferon-gamma (IFN-γ), an important mediator in viral infections, down-regulates PCT production, making PCT a very useful test to help distinguish between viral and bacterial infections.

## Diagnosis

**Fig. 1:**
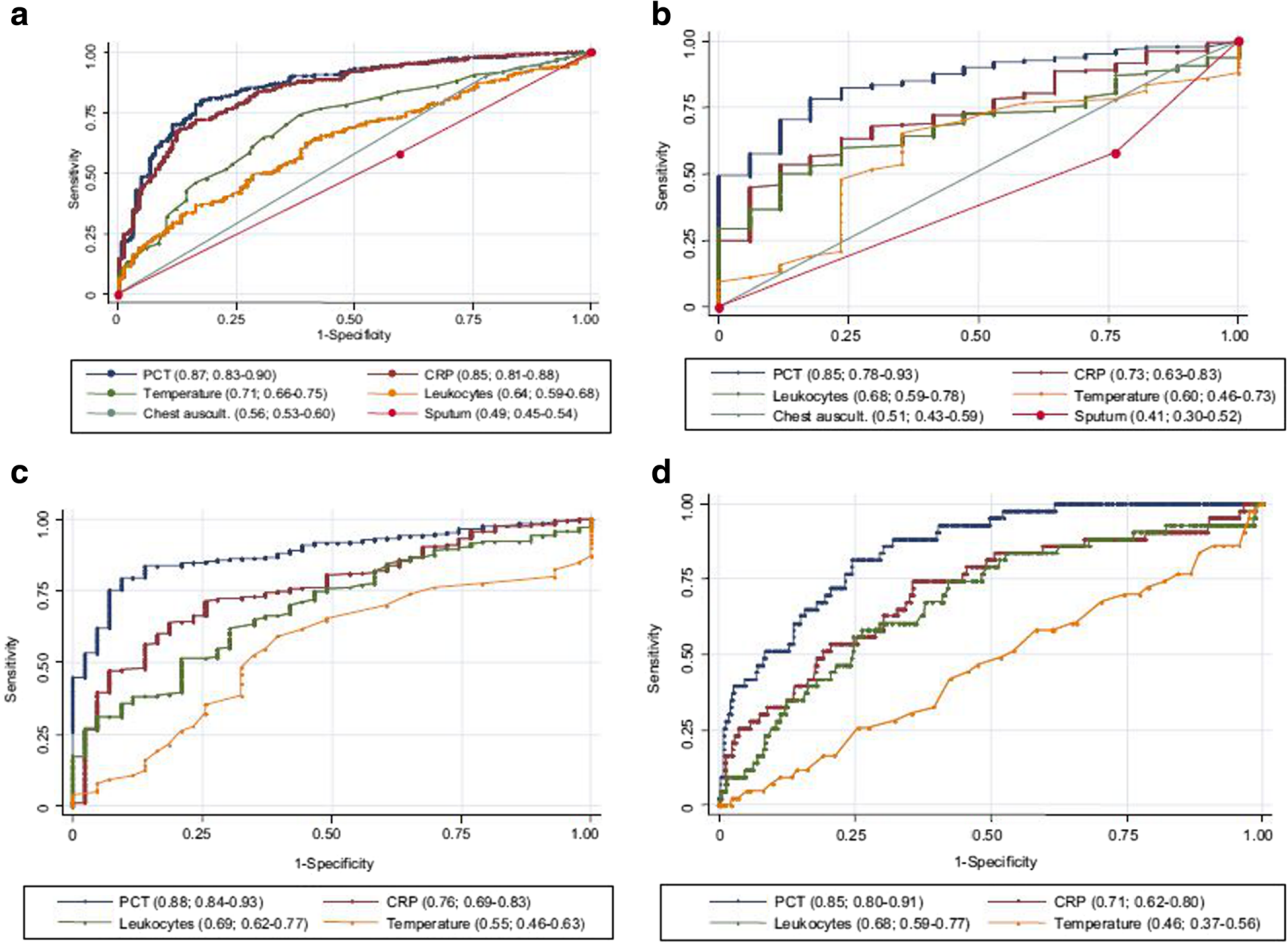
Receiver operating characteristics curves (ROC) of different parameters for the diagnosis of pneumonia. **a** Diagnostic accuracy to predict CAP without chest radiography: Primary care approach. **b** Diagnostic accuracy to predict radiographically suspected CAP (control group (*n* = 20) includes other non-infectious diagnoses initially diagnosed as CAP): Emergency department approach. **c** Diagnostic accuracy to predict radiographically suspected CAP (control group (*n* = 44) includes other non-infectious diagnoses initially diagnosed as CAP (*n* = 20) plus patients without a clinically relevant bacterial aetiology of CAP (*n* = 24). **d** Diagnostic accuracy to predict bacteraemic CAP. Values show areas under the ROC curve with 95% CI. Chest auscult. = abnormal chest auscultation; CRP, C-reactive Protein; PCT, procalcitonin. Sourced from [12]

Biomarkers are helpful in aiding with the initial diagnosis of CAP. In many patients, making the diagnosis of CAP is straightforward: a history is taken, followed by examination and then a chest X-ray to confirm the diagnosis. However, in some patients there might be co-morbidities or the picture might not be typical, which makes it more difficult for the clinician to make a confident diagnosis. As the world’s population ages and the number of patients on therapeutic immunosuppression increases, the differential diagnoses for patients with dyspnea and cough increases. This diagnosis list includes cardiac failure, an acute exacerbation of chronic obstructive pulmonary disease, atypical pneumonia, pulmonary embolism or even interstitial lung disease. This is the place where biomarkers can aid in diagnosis. Müller et al. [12] demonstrated a significant improvement in diagnostic accuracy when adding PCT and CRP to standard clinical signs and symptoms. These biomarkers also performed better than standard makers of infection such as white cell count and raised temperature in different settings, including primary care and the emergency room, as well as in bacteriaemic and non-bacteraemic patients (Fig. 1 [12, 13]). The area under the curve (AUC) of clinical signs and symptoms alone was 0.79 (95% CI 0.75–0.83); with added PCT and highly sensitive CRP it was 0.92 (95% CI 0.89–0.94; *p* < 0.001) [12].

## Response to treatment and antibiotic stewardship

Biomarkers can be monitored to evaluate response to treatment, which can impact length of antibiotic use and predict outcomes. A paper by Coelho et al. [14] adds a new dimension to the basic blood test C-reactive protein (CRP), showing its use to predict outcomes. In patients with severe CAP, they found that a CRP ratio comparing day 5 (D5) CRP against admission CRP predicted ICU outcome, using receiver operating characteristic (ROC) curves with a value of 0.73 (95% CI 0.64–0.82). This prospective observational cohort study classified CAP patients into the following groups: fast response—when D5 CRP was ≤ 0.4 of day 1 (D1) CRP concentration; slow response—when D5 CRP > 0.4 and D7 ≤ 0.8 of D1 CRP concentration; and non-response—when D7 CRP was > 0.8 of D1 CRP concentration. The group then performed comparisons between survivors and non-survivors using standard statistical analysis. When these groups were analysed for ICU survival, mortality was as follows: 4.6% in fast response patients, 17.3% in slow response, and 36.4% in non-response patients (*p* < 0.001). Hospital mortality showed a similar pattern. If clinicians were to use biomarkers in this fashion they could identify patients who are ready for discharge, those who have shown a fast response to treatment versus those patients who might not have adequate source control or resistant organisms; that is, those with slow or no response to treatment who require further interventions to improve outcomes.

**Fig. 2:**
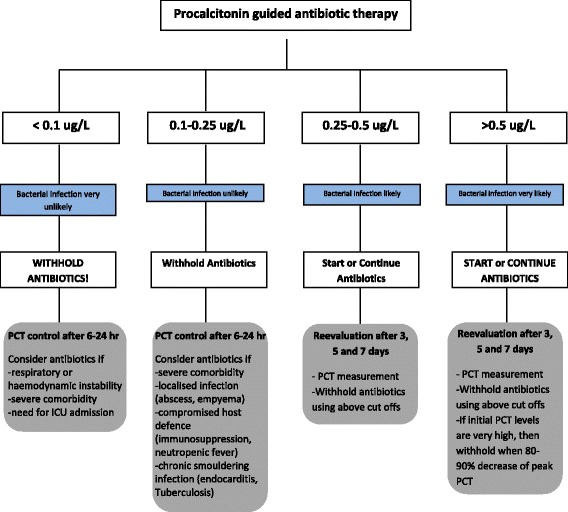
Antibiotic stewardship based on procalcitonin (PCT) cut-off ranges. Re-evaluation of the clinical status and measurement of serum PCT levels is mandatory after 6–24 h in all persistently sick and hospitalized patients in who antibiotic are withheld. PCT, procalcitonin; ICU, intensive care unit. Adapted from [16]

Is it safe to base antibiotic decisions on biomarkers? A large multi-centred, prospective, randomised, controlled, non-inferiority trial [15, 16] with open intervention performed in Switzerland recruited 1359 patients, and was designed to examine whether a PCT algorithm can reduce antibiotic exposure without increasing the risk for serious adverse outcomes (ProHOSP Study). Patients with lower respiratory tract infections (LRTI) presenting to the emergency departments were recruited, including patients with CAP, acute bronchitis and chronic obstructive pulmonary disease with acute exacerbations. The primary non-inferiority end point was a 30-day composite of overall adverse events including death from any cause, ICU admission for any reason, disease specific complications and recurrence of LRTI in need of antibiotics, with or without hospital readmission. Using predetermined PCT cut-offs (Fig. 2 [16],) the trial demonstrated an overall adverse outcome similar in the PCT-driven group versus the control group (15.4% [*n* = 103] vs. 18.9% [*n* = 130]; difference, -3.5%; 95% CI -7.6 to 0.4%). The odds ratio (OR) for the combined adverse outcome was 0.76 (95% CI 0.57–1.01). The secondary endpoint of mean duration of antibiotic exposure was lower in the PCT group versus the control group in all patients (5.7 vs. 8.7 days; relative change, -34.8%; 95% CI -40. to 28.7%) as well as in the subgroup analysis of patients with CAP (*n* = 925, 7.2 vs. 10.7 days, 32.4%; 95% CI -37.6 to -26.9%).

As mentioned previously, IFN-γ down-regulates PCT production, which could help distinguish between viral and bacterial infections. Algorithms based on PCT values have been validated to aid in deciding whether patients have bacterial CAP versus viral CAP and hence require antibiotics. The suggestions are identical to those used in the ProHOSP study above and are as follows: PCT <0.10 mcg/l: strongly discourage antibiotic use; PCT between 0.10-0.25 mcg/l: discourage antibiotic use; PCT between 0.25-0.50 mcg/l: encourage antibiotic use; PCT >0.50 mcg/l: strongly encourage antibiotic use [17]. This has been shown to be effective in studies evaluating LTRI to aid in diagnosis and decrease antibiotic prescription [17, 18]. A recent study [19], which evaluated 2259 adults with radiographic evidence of CAP requiring hospitalisation in the United States, demonstrated that in the 853(38%) patients on whom they were able to culture an organism, 530 (23%) had one or more viruses, 247 (11%) had bacteria, and bacterial and viral pathogens in were found in 59 (3%), The viral burden in CAP is often underappreciated and this study illustrates that PCT may be useful in helping with antibiotic decision making.

The Cochrane meta-analysis evaluating the use of PCT to initiate or discontinue antibiotics in LRTI analysed data from 14 trials with 4221 patients with acute respiratory infections (50% had CAP), including the Swiss cohort. They demonstrated a reduction in antibiotic exposure (from a median of 8 to 4 days) [20]. The reduction of antibiotics used did not impact on outcomes; there was no increase in mortality or treatment failure in any of the clinical settings it was investigated in (outpatient clinic, emergency department) or in patients with any type of acute respiratory infection, including CAP. This can be seen as a major gain for antibiotic stewardship programs.

## Predicting complications, outcomes and mortality

There now is evidence that biomarkers can be also be used to predict complications, outcomes and mortality. A recent study performed in Uganda looking at the usefulness of PCT in HIV-infected individuals with lower respiratory tract infections to predict in-hospital mortality is particularly useful. Often HIV-infected and immunosuppressed patients are excluded from studies; therefore, one is never sure how to extrapolate the biomarker evidence into actual practice, especially if that practice is in a country with a high HIV prevalence. Tokmen and colleagues [21] performed a prospective, nested, case control study on data from the larger International HIV-associated Opportunistic Pneumonias (IHOP) study, investigating the mortality predictability of PCT in HIV-infected individuals with lower respiratory tract infections. This study is different from many of those undertaken before, as it looked at only HIV-infected individuals and at patients with symptoms for longer than 2 weeks, therefore looking to include patients with tuberculosis and Pneumocystis pneumonia (PCP). A cohort of 241 HIV-infected patients had PCT measurements performed. The cohort had advanced HIV disease with a median CD4 count of 47 cells/μL. Unfortunately, a final diagnosis could not be made in 22.8% of the group due to death or inability to follow up; this resulted in a final group of 203 diagnoses in 186 patients. As predicted, tuberculosis was the most common diagnosis at 71.9% (*n* = 146/203), with bacterial pneumonia second at 12.3% (*n* = 25/203) and only 1% being PCP (*n* = 2/203). The median PCT level was 1.45 ng/ml. The authors decided to use a cut-off of 0.5 ng/ml for PCT assessment of mortality prediction. If patients had an elevated PCT (>0.5 ng/ml), it was associated with an increased predicted probability of mortality (1% mortality in those with PCT ≤ 0.5 ng/ml vs. 10% mortality in those with a PCT of > 0.5 ng/ml; *p* = 0.004). This cohort had a reported in-hospital mortality that was very low and this could be a confounding factor if the data were extrapolated to other institutions. They performed further data analysis and found that by combining elevated PCT plus tachypnoea plus hypoxaemia, the accuracy of in-hospital mortality prediction resulted in an AUC of 0.741 (95% CI 0.65–0.83). This was a marked improvement compared to tachypnoea and hypoxaemia without PCT, which had an AUC of 0.659 (95% CI 0.55–0.77; *p* = 0.05). It is exciting that the authors have shown that adding a biomarker to easily assessable clinical criteria can markedly improve the ability to predict in-hospital mortality.

Another study [22], from the large Swiss cohort previously mentioned, evaluated the performance of PCT as a prognostic indicator, alongside the already validated CAP scores, the PSI, and the CURB-65 score. The study of the prognostic potential of PCT was a predefined secondary end point of the large multicentre Procalcitonin Guided Antibiotic Therapy and Hospitalisation in Patients with Lower Respiratory Tract Infections (ProHOSP) study. A total of 925 patients were included in the analysis. The median age of patients enrolled was 73 years, with 41% being female. While this population group would be appropriate for the developed world, those who practice in the developing world where the population would be much younger should take this into consideration. Survivors had a much lower median PCT level on admission than non-survivors: 0.44 ug/l (IGR 0.15–2.63 ug/l) versus 0.83 ug/l (IQR 0.30–5.67 ug/l) (*p* = 0.02), as did patients without adverse outcomes versus those with adverse outcomes: 0.39 ug/l (IQR 0.14–2.2 ug/l) vs. 1.30 ug/l (IQR 0.38–7.47 ug/l); *p* < 0.001. This group did not find that PCT improved the AUC to predict morality (AUC 0.60), and was in fact lower than the clinical risk scores alone (AUC CURB-65 = 0.72; AUC PSI = 0.79). Even when PCT was added to the PSI and CURB-65 clinical scores, it did not significantly improve either for mortality prediction. However, for prediction of adverse events combining the PCT with either the CURB-65 or PSI improved the ability to predict these events better than the clinical scores alone (PSI plus PCT AUC = 0.71 [0.66–0.76], *p* < 0.01; CURB-65 plus PCT AUC = 0.70 [0.65–0.75], *p* = 0.008). This group also found that the follow up PCT levels measured on day 3, 5 and 7 were able to provide information about increased risk of mortality and adverse events in a very similar fashion to the Coelho group [17]. Those patients who did not show a clear decrease of PCT had worse outcomes.

Skouras and colleagues [23] used the biomarker CRP to evaluate its clinical utility as a predictor in parapneumonic effusions (PPE). It has been estimated that approximately 40% of CAP can be associated with a PPE, most of which are inconsequential [24]. Up to 7.2% of CAP patients can go on to develop complicated parapneumonic effusions (CPPE) or empyema [25]. This is a significant burden in CAP patients and having the ability to predict or be aware of patients who are likely to develop this complication would be very useful. This was a prospective study from two tertiary Greek hospitals and 54 patients were included. Whilst this a small sample size, there is very little evidence-based medicine in this field. The cohort was evaluated in 2 groups, those with uncomplicated PPE and those with CPPE. Admission serum and pleural CRPs were compared in these 2 groups, as well as residual pleural thickening 6 months after hospital discharge. Residual pleural thickening can result in restrictive pulmonary disease. This study did demonstrate that an elevated serum CRP of >150 mg/l had a sensitivity of 61% and specificity of 91%, with a ROC of 0.82, for predicting residual pleural thickening post-PPE. The higher CRP levels are a marker of a more exuberant inflammatory response, which could explain the greater risk for pleural thickening. This finding adds a useful and easy test to allow for identification of patients who are at risk for long-term complications from PPE. Serum CRP and pleural CRP were significantly higher in CPPE. ROC curves evaluating accuracy to identify CPPE were 0.73 for pleural CRP and 0.68 for serum CRP, compared to the ROC of the more traditionally used low pleural fluid pH (pH < 7.1) of 0.93. Therefore, by themselves these tests are clearly inferior to tests used in current clinical practice. However, when combined with the classical criteria using an ‘AND’ or ‘OR’ rule, the positive and negative predictive values for diagnosis of a CPPE were improved. The group concluded that the serum CRP has value as an independent predictor for the development of residual pleural thickening. They also showed that it could be used in combination with the more traditional biomarkers—pleural fluid LDH, glucose and pH—to help with treatment decisions in non-purulent PPE.

## The future

The biomarker of the future will most likely be pro-adrenomedullin (pro-ADM). Adrenomedullin (ADM) is produced at times of physiologic stress and has vasodilatory, antimicrobial, and anti-inflammatory properties. As early as 1996, Hirata and colleagues showed that levels of ADM increased with disease severity in adults with sepsis [26]. However ADM is not an ideal biomarker as it is rapidly cleared from circulation due to its rapid binding to receptors and its half-life of 22 min, so midregional-proadrenomeddullin (MR-proADM), a more stable precursor molecule, is used in clinical practice.

There have been several studies that have shown that MR-proADM has better predictive power than even CRP and PCT. In a prospective cohort study of 491 patients with CAP, admission MR-proADM levels correlated with and improved clinical severity scores [27]. Studies by Christ-Crain et al. and Kruger et al. both demonstrated the improved mortality prediction of MR-proADM when added to the PSI [28, 29]. A systematic review, which included twelve studies, evaluated the prognostic value of MR-proADM in short and long term mortality in CAP. The authors demonstrated an increase in short-term mortality (OR = 6.8; 95% CI: 4.65–10.13; *p* < 0.001) and complications (OR = 5.0; 95% CI: 3.86–6.49; *p* < 0.001) with elevated MR-proADM. A further pooled analysis of 4 of the studies showed an improved discriminant ability for predicting mortality in CAP patients of 8% (95% CI: 2–14%) when MR-proADM was added to the CURB-65/CRB-65 score [30]. When this test becomes more commercially available, there will be another important tool for CAP outcome prediction.

Further fascinating work has recently been done to identify a novel biomarker in CAP by analysis of the blood genomic response in CAP patients. Global gene expression profiles of whole blood leukocytes were collected within 24 h after ICU admission from CAP and non-CAP patients and then compared with those of healthy individuals [31]. A 78-gene signature was defined for CAP and the FAIM3:PLAC8 gene expression ratio was derived with an AUC of 0.845 (95% CI, 0.764–0.917) and positive and negative predictive values of 83 and 81%, respectively, when looking at ability to diagnosis CAP. Interestingly FAIM3, encoding the FAS apoptotic inhibitory molecule 3, and PLAC8, encoding placenta-specific 8, are both negative regulators of apoptosis. The FAIM3:PLAC8 ratio outperformed plasma procalcitonin and IL-8 and IL-6 in discriminating between CAP and non-CAP patients. Studies looking at gene expression in CAP, such as the above by Scicluna and colleagues, are not only about biomarkers but also add information to the understanding of the pathophysiology of the immune response in CAP.

Another up and coming approach to the biomarker field is that of metabolomics. Metabolomics is the investigations of the biochemical molecules derived from cellular processes, and it is studied under specific conditions, including CAP [32]. An example of metabolomics application in CAP is kynurenine (Kyn). Kyn is a toxic metabolite formed during the degradation of tryptophan (Trp), and patients with CAP and sepsis show significantly higher Kyn levels and lower Trp levels compared to controls [33, 34]. A more familiar metabolomic example, which has been used in critical care for prognosis and severity, is lactate. In adults with pneumonia, the lactate level has been shown to be a better predictor of 28-day mortality than the CURB-65 score, and a combination of CURB-65 with lactate level improves the predictive value of CURB-65 score alone [35]. For further reading Nickler and colleagues have written an interesting review article looking at this growing field and its impact on our understanding of respiratory conditions [32].

## Conclusion

Point-of-care tests are now available, making it possible to utilise knowledge about biomarkers, even in the emergency room and doctor’s room setting. Clinicians can make real-time decisions about the care and the admission path of patients. Once there is a complete understanding of all the information that can be gained from these simple tests, there will be greater insight into the future management of patients with CAP. This is especially true with regards to predicting complications and outcomes and, most importantly, response to treatment and earlier discontinuation of antibiotics. Hopefully, in the future, the benefits of implementing what is known about biomarkers will be reflected in improved patient outcomes.

## Abbreviations

ADM: Adrenomedullin
AUC: Area under the curve
CAP: Community acquired pneumonia
CPPE: Complicated parapneumonic effusion
CRB-65: Confusion, Respiratory rate, Blood pressure, age > 65
CRP: C-reactive protein
CURB-65: Confusion, Urea, Respiratory Rate, Blood pressure, age > 65
ESR: Erythrocyte sedimentary rate
FAIM3: FAS apoptotic inhibitory molecule 3
HIV: Human immunodeficiency virus
IFN-γ: Interferon gamma
IL: Interleukin
Kyn: Kynurenine
LRTI: Lower respiratory tract infection
MR-proADM: Midregional-proadrenomeddullin
OR: Odds ratio
PAMPS: Pathogen associated molecular pattern
PCP: Pneumocystis pneumonia
PCT: Procalcitonin
PLAC8: Placenta-specific 8
PPE: Parapneumonic effusion
pro-ADM: Pro-adrenomedullin
ROC: Receiver operating curve
TNF-α: Tumour necrosis factor alpha
Trp: Tryptophan
WCC: White cell count
